# Comprehensive Analysis of the Immune Implication of *ACK1* Gene in Non-small Cell Lung Cancer

**DOI:** 10.3389/fonc.2020.01132

**Published:** 2020-07-23

**Authors:** Jinhong Zhu, Yang Liu, Haijiao Ao, Mingdong Liu, Meng Zhao, Jianqun Ma

**Affiliations:** ^1^Department of Clinical Laboratory, Biobank, Harbin Medical University Cancer Hospital, Harbin, China; ^2^Department of Clinical Oncology, Harbin Medical University Cancer Hospital, Harbin, China; ^3^Department of Thoracic Surgery, Harbin Medical University Cancer Hospital, Harbin, China

**Keywords:** ACK1, immune cells, NSCLC, prognosis, nomogram

## Abstract

Activated Cdc42-associated kinase1 (ACK1), a non-receptor tyrosine kinase, has been considered as an oncogene and therapeutic target in various cancers. However, its contribution to cancer immunity remains uncertain. Here we first compared the profiles of immune cells in cancerous and normal tissues in The Cancer Genome Atlas (TCGA) lung cancer cohorts. Next, we found that the immune cell infiltration levels were associated with the *ACK1* gene copy numbers in lung cancer. Consistently, our RNA-seq data unveiled that the silencing of *ACK1* upregulated several immune pathways in lung cancer cells, including the T cell receptor signaling pathway. The impacts of ACK1 on immune activity were validated by Gene Set Enrichment Analysis of RNA-seq data of 188 lung cancer cell lines from the public database. A pathway enrichment analysis of 35 ACK1-associated immunomodulators and 50 tightly correlated genes indicated the involvement of the PI3K-Akt and Ras signaling pathways. Based on ACK1-associated immunomodulators, we established multiple-gene risk prediction signatures using the Cox regression model. The resulting risk scores were an independent prognosis predictor in the TCGA lung cohorts. We also accessed the prognostic accuracy of the risk scores with a receiver operating characteristic methodology. Finally, a prognostic nomogram, accompanied by a calibration curve, was constructed to predict individuals' 3- and 5-year survival probabilities. Our findings provided evidence of ACK1's implication in tumor immunity, suggesting that ACK1 may be a potential immunotherapeutic target for non-small cell lung cancer (NSCLC). The nominated immune signature is a promising prognostic biomarker in NSCLC.

## Introduction

Lung cancer is the most frequently diagnosed cancer and one of the most lethal solid malignancies (1). Non-small cell lung cancer (NSCLC), the primary histology type, constitutes 85% of all cases. Despite considerable progress and advances in the understanding of lung cancer biology and multimodality treatments, prognosis remains far from satisfying. Patients with unresectable advanced diseases only receive modest benefits from typical therapies, including systemic cytotoxic chemotherapy, radiation therapy, as well as targeted therapies against epidermal growth factor receptor and anaplastic lymphoma kinase fusion oncogene (2). Fortunately, immunotherapy has been emerging as a promising alternative treatment for some lung cancer patients. Evading immune destruction is one of 10 putative cancer hallmarks (3). In several clinical trials, a fraction of patients achieved a partial response after receiving immune checkpoint inhibitors that enhance the cytotoxic activity of immune effector cells by neutralizing cytotoxic T-lymphocyte antigen-4 (CTLA4), programmed cell death protein 1 (PD-1), or PD-1 ligand (PD-L1) (2). Several checkpoint blockers (nivolumab, pembrolizumab, and atezolizumab) have been proven to treat NSCLC patients (4, 5). However, like other therapies, only a portion of NSCLC patients benefit from immunotherapy. Prognostic immune markers would facilitate the identification of the subgroups responding to immunotherapies. Some evidence has suggested that tumor-infiltrating leukocytes are related to the clinical response to therapy and cancer prognosis, including NSCLC (6–10). However, the molecular features designating an intratumoral immune microenvironment remain to be explored at length. Therefore, it is indispensable to comprehensively understand lung cancer immunology and the molecular regulatory mechanisms to ensure the success of immunotherapy.

Activated Cdc42-associated kinase1 (ACK1), referred to as tyrosine kinase non-receptor 2 (TNK2), is an intracellular non-receptor tyrosine kinase. The *ACK1* gene located on chromosome 3q29 is frequently amplified or mutated in several types of cancers, which generally leads to an abnormal activity of the ACK1 signaling cascades (11). ACK1 is a promising therapeutic target in cancer, including colorectal cancer (12), breast cancer, hepatocellular carcinoma (13), gastric cancer (14–16), ovarian cancer, and NSCLC (17, 18). Thus far, the mechanisms underpinning the oncogenic role of ACK1 in lung cancer remain mostly unknown (17). Intriguingly, our preliminary study revealed that ACK1 is involved in the control of several immune-related pathways. The immune implication of ACK1 in cancer has not been reported so far. Here we systematically evaluated the status of lymphocytes and clarified the association between ACK1 and lung cancer immunity, as well as the signaling pathways regulating the ACK1-mediated immune response. Finally, we generated prognostic immune signatures using ACK-associated immunomodulators, followed by the construction of a nomogram by integrating the immune signature and other clinical features.

## Methods and Materials

### Acquirement of NSCLC Expression Profiles From TCGA Datasets

We obtained LUAD and LUSC datasets from the TCGA project (https://portal.gdc.cancer.gov/). All the RNA-Seq data (level 3) were normalized as fragments per kilobase of transcript per million mapped reads. The LUAD dataset contained 535 cancerous and 59 normal tissues, while the LUSC dataset comprised 502 cancerous and 49 normal tissues, accompanied by clinical information. The voom function in the limma package for R software was employed to further process RNA expression data.

### Determination of Tumor-Infiltrating Immune Cells in TCGA Lung Cancer

We adopted Cell type Identification By Estimating Relative Subsets Of RNA Transcripts (CIBERSORT) method to qualify and quantify 22 types of immune cells in tissues, including seven T cell types, naïve and memory B cells, plasma cells, NK cells, and myeloid subsets (10, 19). CIBERSORT was developed to identify cell types by using the signature gene expression profile for the high-throughput array or RNA-sequencing data (10, 19). This method mainly relies on a leukocyte gene signature matrix, called LM22. This file comprises 547 genes that differentiate 22 human hematopoietic cell phenotypes (19). With the CIBERSORT L22 as the reference, we analyzed the mRNA expression matrix using CIBERSORT R script acquired from the CIBERSORT website (https://cibersort.stanford.edu/). By using Monte Carlo sampling (20), we calculated an empirical *P*-value for the deconvolution of each case. After excluding samples with *P* ≥ 0.05, the following samples were included in the study: 511 LUAD vs. 58 normal samples and 413 LUSC vs. 49 normal samples.

### Correlation Between ACK1/TNK2 and Tumor Immune Cell Infiltration

Tumor Immune Estimation Resource is a web server providing a comprehensive analysis of tumor immune cells of pan-cancer (cistrome.dfci.harvard.edu/TIMER/) (21). By taking advantage of this web tool, we accessed six immune infiltrates (B cells, CD4+ T cells, CD8+ T cells, neutrophils, macrophages, and dendritic cells) in LUAD and LUSC. Several modules were offered on this website, including Gene, Survival, Mutation, SCNA, Diff Exp, Correlation, and Estimation. Relations between immune cell infiltration and survival, *TNK2* copy numbers, and infiltration levels, as well as *ACK1*/*TNK2* mRNA expression levels and individual immune cell infiltration, were explored.

### Cell Culture and RNA-seq

Human lung adenocarcinoma cell line A549 was provided by the Chinese Academy of Sciences Collection Committee cell bank (Shanghai, China). The cells were incubated in an incubator at 37°C, supplied with 5% CO_2_. We knocked down the *ACK1* gene by infecting A549 cells with lentiviral vectors encoding shRNA against *ACK1* (tgCTTCCTCTTCCACCCAATT) or shRNA control vectors purchased from the GeneChem (Shanghai, China). The RNA samples extracted from cell cultures (shACK1 vs. shcontrol) in triplicate were subjected to RNA-seq using the BGISEQ-500 platform.

### Gene Set Enrichment Analysis

The Cancer Cell Line Encyclopedia (CCLE) project was launched and maintained by the Broad Institute, the Novartis Institutes for Biomedical Research, and its Genomics Institute of the Novartis Research Foundation. This database involves the exhaustive expression data of 84,433 genes of 1,457 cell lines, including copy number, mRNA expression (Affymetrix), RPPA, RRBS, and mRNA expression (RNAseq) (3). This project covers most of the common cancer types, such as breast cancer, stomach cancer, kidney cancer, liver cancer, and lung cancer. We downloaded and processed the RNAseq data of 188 lung cancer cell lines from the CCLE website. *ACK1*/*TNK2* expression profiles were extracted for subsequent analysis. The average expression of the *ACK1*/*TNK2* gene acted as a cutoff value to split all lung cancer cell lines into *ACK1*/*TNK2* ^high^ and *ACK1*/*TNK2* ^low^ groups. Gene set enrichment analysis (GSEA) (22, 23) was employed to dissect the signaling pathways significantly associated with ACK1 expression levels (*ACK1*/*TNK2* ^high^ vs. *ACK1*/*TNK2* ^low^). GSEA software 4.0.0 was downloaded for the analysis (software.broadinstitue.org/gsea/index.jsp).

All genes were ranked concerning their differential expression between two phenotypic groups. GSEA assesses the genome-wide expression profiles at the levels of gene sets instead of concentrating on only a handful of mostly altered genes. A gene set denotes a collection of concordant genes that have a similar biological function, chromosomal location, or regulation. The Molecular Signatures Databases were predetermined for use with GSEA software according to known biological knowledge, including cellular processes and biochemical pathways (e.g., cell cycle, DNA replication, apoptosis, and mTOR signaling pathway). If the members of a predefined gene set mainly hit the top or the bottom of, but not randomly scattered through, the ranked gene list, this gene set is thought to be associated with the phenotypic difference (22, 23).

### Immunomodulators

Immunomodulators associated with ACK1 were retrieved from an online integrated database TISIDB (http://cis.hku.hk/TISIDB/), aiming to elucidate tumor–immune system interactions (24). This web portal was built based on data collected and integrated from the following resources: PubMed database, high-throughput screening data investigating the responses of tumor cells to T cytotoxic cells, exome and RNA sequencing data of patients receiving immunotherapy, TCGA, and other public databases [e.g., Uniprot, Gene Ontology (GO), and DrugBank]. We chose immunoinhibitors and immunostimulators that were significantly correlated with ACK1 regarding gene expression (Spearman correlation test, *P* < 0.05). Next, we uploaded the ACK1-associated immunomodulators onto the cBioPortal for Cancer Genomics (www.cbioportal.org). With the network module, the queried genes were able to seize 50 concomitantly altered genes based on the RNA-seq data of cancer samples. The resulting protein network was subjected to GO annotation and Kyoto Encyclopedia of Genes and Genomes (KEGG) pathway enrichment analysis using two web-based tools (https://string-db.org/) and WEB-based GEne SeT AnaLysis Toolkit (http://www.webgestalt.org/) (2, 25).

### Survival Analysis

We attempted to develop a prognostic multiple immune gene signature out of ACK1-associated immunomodulators. The stepwise variable selection was performed with the Akaike Information Criterion in Cox models (26). After the immune genes were chosen, the prognostic index, referred to as risk score, was generated: risk score = β_1_*x*_1_ + β_2_*x*_2_ +… + β_*i*_*x*_*i*_. In this formula, *x*_*i*_ was the expression level of each gene, while β_*i*_ is the risk coefficient of each gene derived from the Cox model (27). Kaplan–Meier survival curve, log-rank test, as well as univariate Cox analyses were adopted to appraise the association of the immune-related gene signature and clinical characteristics with overall survival. Multivariate analysis was performed for the risk score with adjustment for age, sex, T, N, M, and TNM stage. The time-dependent receiver operating characteristic (ROC) curves were adopted to determine the prognostic accuracy of the risk score using the survivalROC package (28).

### Construction of Nomogram

Since its development, nomograms have been increasingly used for predicting cancer prognosis. This statistical methodology scores each contributing parameter by points, such as age, gender, TNM stage, and risk score. For each individual, all these points are added up to generate a total point, with larger points meaning that an event has a higher chance to occur. This method allows the personalized estimate of the probability of recurrence, death, or drug adherence (29). In this study, we used the nomogram for cancer prognosis by incorporating the clinical characteristics and the risk scores of patients. The normogram was created via the rms package for R software. With the application of the bootstrap method (1,000 replicates), a calibration curve was used to visualize the deviation of predicted probabilities from what actually happened. The concordance index (C-index) was used to measure the predictive accuracy of the nomogram.

### Statistics

We implemented all statistical analysis with R version 3.5.0 (R Foundation for Statistical Computing, Vienna, Austria), complemented by IBM SPSS Statistics 24.0 (IBM, Inc., Armonk, NY, USA). One-way ANOVA was used to perform a comparison for continuous variables among groups ≥3, followed by *post-hoc* tests. The threshold of statistical significance was set as *P* < 0.05.

## Results

### The Landscape of Infiltrating Immune Cells in Lung Cancers and Normal Tissues

**Figure 1:**
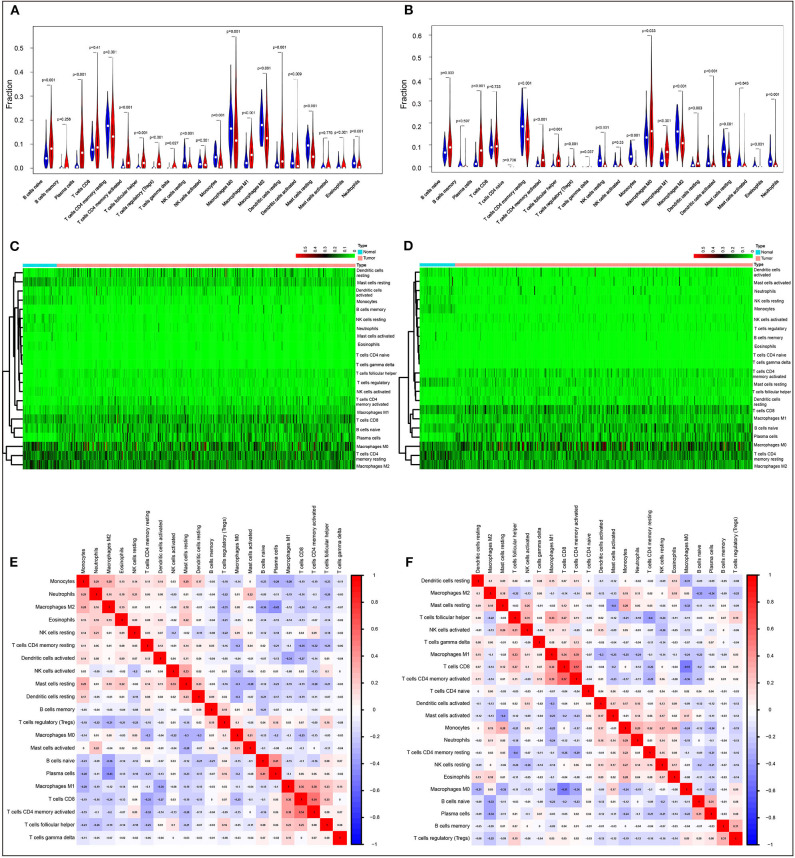
Evaluation of the proportions of 22 types of immune cell infiltration by Cell Type Identification By Estimating Relative Subsets Of RNA Transcripts method in The Cancer Genome Atlas lung cancer cohorts. Violin plots and heatmaps indicated the differences in the immune cell distribution between malignant (red) and normal (blue) tissues in LUAD **(A,C)** and LUSC **(B,D)** cohorts. Different correlation patterns among 26 immune cell subsets in LUAD **(E)** and LUSC **(F)** cohorts.

We first systematically delineated the pattern of immune cells by extracting and processing the signature gene expression profile with the CIBERSORT method. After removing the samples with *P* ≥ 0.05, the landscape of the infiltrating immune cells in cancerous and healthy biopsies for TCGA lung carcinoma (LUAD) and lung squamous carcinoma (LUSC) cohorts is displayed in Supplementary Figures 1A,B, respectively. When compared to normal tissues, the proportions of naïve B cells, plasma cells, T cells CD8, T cells CD4 memory activated, T cell follicular helper, T cells regulatory (Tregs), T cells gamma delta, macrophages M1, and dendritic resting cells were significantly increased, while T cells CD4 memory resting, NK cells resting, monocytes, macrophages M0, macrophages M2, activated dendritic cells, resting mast cells, eosinophils, and neutrophils in LUAD decreased (Figure 1A). A similar situation was found for LUSC (Figure 1B). In comparison to normal tissues, different patterns of the infiltrating immune cells LUAD and LUSC are shown in Figures 1C,D, respectively. Moreover, different correlation patterns among the immune cells were found in LUAD (Figure 1E) and LUSC (Figure 1F), suggesting the different immune microenvironment between these two histology types. Besides that, the infiltration levels of B cells and dendritic cells, as well as *ACK1*/*TNK2* mRNA expression, were significantly associated with survival in LUAD (Supplementary Figure 2A), whereas CD4^+^ T cells were associated with survival in LUSC (Supplementary Figure 2B).

### Association Between *ACK1*/*TNK2* and Immune Cells

**Figure 2:**
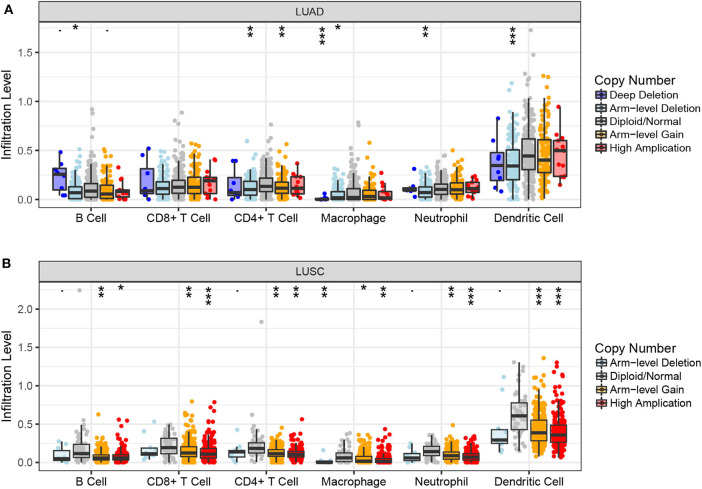
Associations between *ACK1*/*TNK2* gene copy numbers and immune cell infiltration levels. Association between *ACK1*/*TNK2* copy numbers and immune cell infiltration levels in LUAD **(A)** and LUSC **(B)** cohorts. *p < 0.05; **p < 0.01; ***p < 0.001.

**Figure 3:**
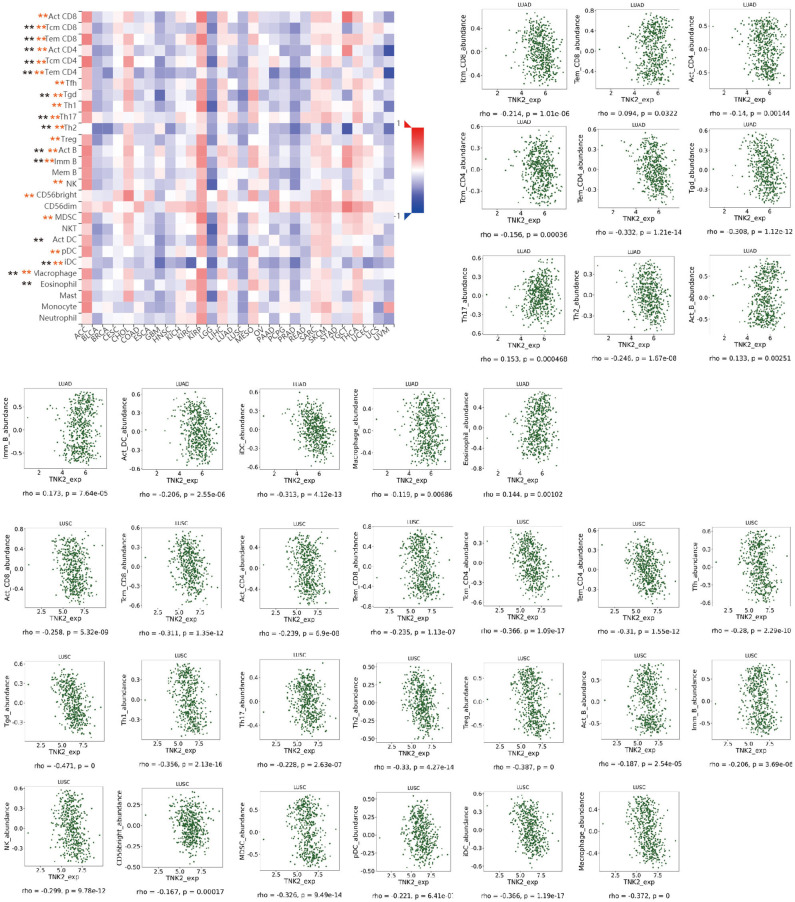
Correlation between *ACK1*/*TNK2* expression levels and immune cell subsets. The black and red asterisks in the correlation heatmap indicated immune cell types significantly associated with *ACK1*/*TNK2* expression levels in LUAD and LUSC cohorts, respectively. The dot plots displayed the correlations between *ACK1*/*TNK2* expression levels and immune cell subsets in LUAD (upper panel) and LUSC (low panel). *p < 0.05; **p < 0.01.

Next, we examined the impact of ACK1/TNK2 on the immune systems. *ACK1*/*TNK2* mRNA expression levels were significantly elevated in many types of cancers, including LUAD and LUSC (Supplementary Figure 3). The immune cell infiltration levels changed along with the *ACK1*/*TNK2* gene copy numbers. Several immune cell infiltration levels seemed to associate with altered *ACK1*/*TNK2* gene copy numbers, including B cell, CD4+T cell, macrophage, neutrophil, and dendritic cell in LUAD (Figure 2A). Similar results were found in LUSC (Figure 2B). In LUAD, some immune subsets were either negatively or positively associated with *ACK1*/*TNK2* mRNA levels (Figure 3). In contrast, the *ACK1*/*TNK2* mRNA levels were uniformly negatively correlated to various types of immune cells in LUSC (Figure 3).

**Figure 4:**
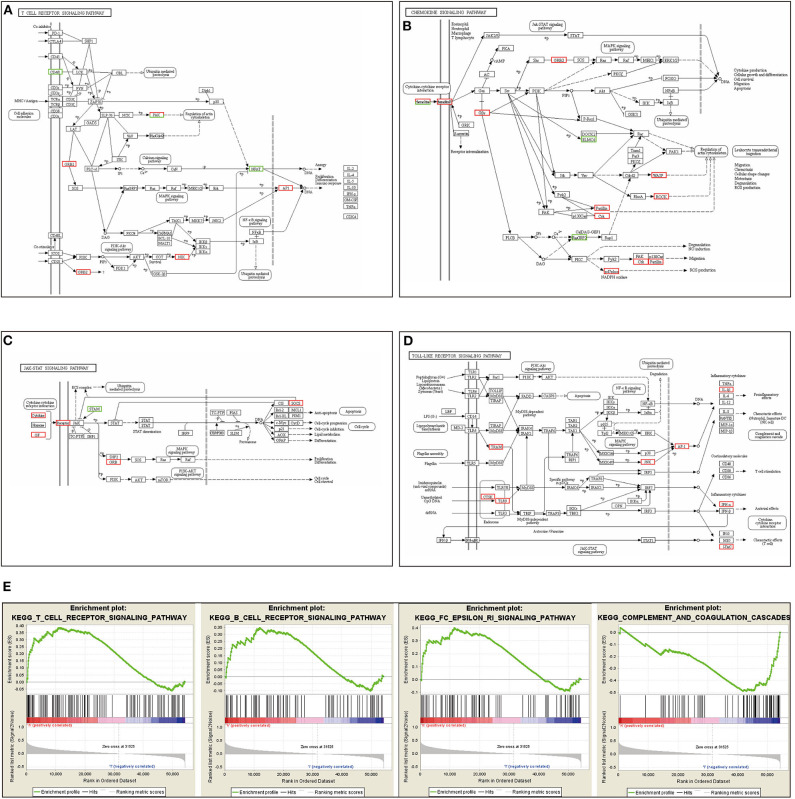
Immune signaling pathways related to ACK1/TNK2. **(A–D)** RNA-seq analysis after the silencing of the *ACK1*/*TNK2* gene of A549 cells. Differentially expressed genes were involved in the T cell receptor signaling pathway, chemokine signaling pathway, JAK-STAT signaling pathway, and Toll-like signaling pathways. **(E)** Dissection of ACK1-associated immune signaling pathways by Gene Set Enrichment Analysis of 188 lung cancer cell lines from the Cancer Cell Line Encyclopedia database.

Our RNA-seq results revealed that the silencing of the *ACK1* gene in A549 cells activated several immune-related signaling pathways, including the T cell receptor, chemokine, JAK-STAT, and Toll-like receptor signaling pathway (Figures 4A–D). These results further confirmed that ACK1 might downregulate the immune reaction of lung adenocarcinoma. We also analyzed the RNA-seq data of 188 lung cancer cells downloaded from the CCLE. The cell lines were dichotomized into *ACK1*^high^ and *ACK1*^low^ groups by the average *ACK1* mRNA level. As shown in Figure 4E, GSEA analysis demonstrated that ACK1 was associated with several immune-associated signaling pathways, including T cell receptor signaling pathway (NES = 1.53, *P* = 0.019), B cell receptor signaling pathway (NES = 1.34, *P* = 0.069), FC epsilon RI signaling pathway (NES = 1.52, *P* = 0.016), and complement and coagulation cascades (NES = −1.55, *P* = 0.019).

**Figure 5:**
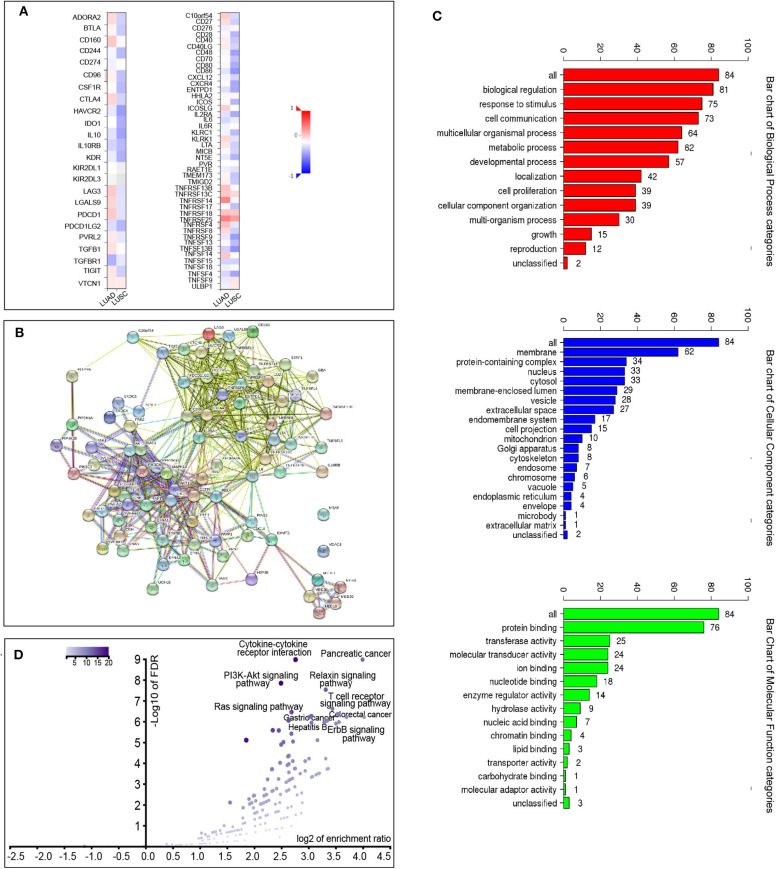
Identification and analysis of immunomodulators associated with the *ACK1*/*TNK2* gene. **(A)** The heatmaps of correlation between the immunoinhibitors and the *ACK1*/*TNK2* gene (left panel) in LUAD and LUSC; the heatmap of correlation between the immunostimulators and the *ACK1*/*TNK2* gene in LUAD and LUSC (right panel). **(B)** Protein–protein network of 35 ACK1-associated immunomodulators and 50 closely related genes in LUAD, produced by the STRING online server. **(C)** Gene Ontology annotation of 35 ACK1-associated immunomodulators and 50 closely connected genes in LUAD. **(D)** Kyoto Encyclopedia of Genes and Genomes pathway analysis of the abovementioned 85 genes.

We also explored the signaling pathways by which ACK1 might modulate the immune response in LUAD. We identified 20 immunostimulators (C10orf54, CD27, CD86, ENTPD1, ICOSLG, IL2RA, IL6, LTA, TNFRSF13B, TNFRSF13C, TNFRSF14, TNFRSF18, TNFRSF25, TNFRSF4, TNFRSF8, TNFRSF9, TNFSF13B, TNFSF14, TNFSF15, and TNFSF4) (Figure 5A) and 15 immunoinhibitors (ADORA2A, CD160, CTLA4, HAVCR2, IL10, IL10RB, LAG3, LGALS9, PDCD1, PDCD1LG2, PVRL2, TGFB1, TGFBR1, TIGIT, and VTCN1) (Figure 5A) significantly associated with ACK1 in LUAD. We queried 50 top genes that were tightly correlated to these immunomodulators using the cBioPortal for Cancer Genomics (Figure 5B). GO was used to annotate these genes (Figure 5C). The KEGG pathway enrichment analysis of these genes indicated that PI3K-AKT, Ras, and T cell receptor signaling pathways were related to ACK1-mediated immune events (Figure 5D).

### The Prognostic Implication of ACK1-Associated Immunomodulator in Lung Cancer

**Figure 6:**
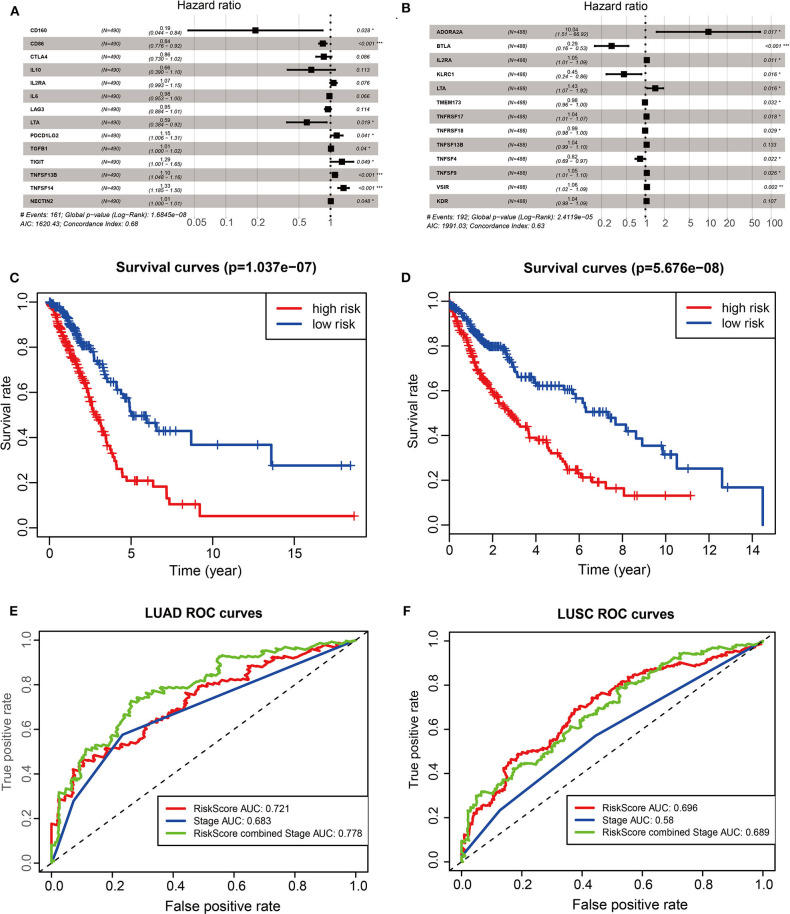
The development of prognostic gene signatures based on 35 ACK1-associated immunomodulators and ACK1. The hazard ratios of genes integrated into the prognostic signatures are shown in the forest plots for LUAD **(A)** and LUSC **(B)**. Kaplan–Meier curves for LUAD **(C)** and LUSC **(D)** regarding the risk scores. Time-dependent receiver operating characteristic curves at 5-years for LUAD **(E)** and LUSC **(F)**.

**Figure 7:**
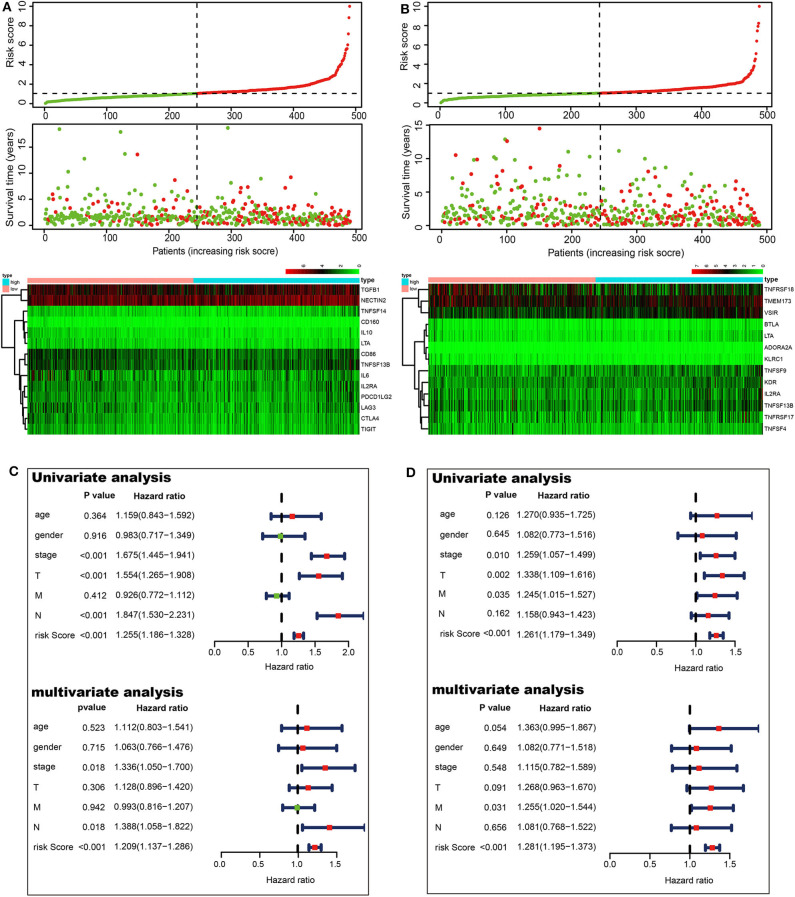
Prognostic values of the risk scores in The Cancer Genome Atlas lung cancer cohorts. Distribution of risk scores, along with survival statuses, and gene expression profiles for LUAD **(A)** and LUSC **(B)**. Univariate and multivariate Cox regression analyses of the risk score in LUAD **(C)** and LUSC **(D)** regarding overall survival.

To investigate the prognostic values of ACK1-associated immunomodulators in LUAD, we entered these variables into a stepwise multivariate Cox regression analysis. This method led to an optimal 14-gene prognostic signature in LUAD. The biological functions of genes integrated into the signature are presented in Table 1. The univariate Cox regression analysis was performed to evaluate the association between these genes and OS (Figure 6A). The risk scores were calculated by adding up the product of expression value and coefficient of each gene. The Kaplan–Meier survival curve elucidated that patients with low-risk scores had significantly longer survival than those with high risk (log-rank test, *P* < 0.001) (Figure 6C). The area under the curve (AUC) values of the risk score and stage were 0.721 and 0.683, respectively. An AUC of 0.778 was achieved when the risk score and stage were combined (Figure 6E). The distribution of risk scores, survival statuses, and signature gene expression profiles for LUAD are visualized in Figure 7A. As seen in Figure 7C, the risk score was significantly associated with survival in TCGA-LUAD in the univariate COX regression model [hazard ratio (HR) = 1.255, 95% confidence interval (CI) = 1.186–1.328, *P* < 0.001]. Moreover, multivariate Cox regression demonstrated that the risk score was an independent predictor of prognosis in NSCLC after adjusting for age, gender, stage, T, M, and N (HR = 1.209, 95% CI = 1.137–1.286, *P* < 0.001). Similarly, 35 ACK1-associated immunostimulators (C10orf54, CD27, CD28, CD40, CD40LG, CD48, CD70, CD80, CD86, CXCL12, CXCR4, ENTPD1, ICOS, IL2RA, KLRC1, KLRK1, LTA, MICB, NT5E, RAET1E, TMEM173, TMIGD2, TNFRSF13C, TNFRSF17, TNFRSF18, TNFRSF25, TNFRSF4, TNFRSF8, TNFRSF9, TNFSF13, TNFSF13B, TNFSF15, TNFSF4, TNFSF9, and ULBP1) and 19 immunoinhibitors (ADORA2A, BTLA, CD96, CD244, CSF1R, CTLA4, HAVCR2, IDO1, IL10, IL10RB, KDR, LAG3, LGALS9, PDCD1, PDCD1LG2, PVRL2, TGFBR1, TIGIT, and VTCN1) were acquired for LUSC. With these immunomodulators, we generated a 13-gene prognostic signature in LUSC (Figure 6B; Table 1). The risk score was significantly associated with survival in LUSC as indicated by the Kaplan–Meier survival curve (log-rank test, *P* < 0.001), ROC (combined AUC = 0.696), as well as univariate (HR = 1.261, 95% CI = 1.179–1.349, *P* < 0.001) and multivariate (HR = 1.281, 95% CI = 1.195–1.373, *P* < 0.001) Cox regression analyses (Figures 6D,F, 7B,D).

**Table 1:** Functions of the genes included in the prognostic signatures.

**LUAD**			**LUSC**		
**Gene symbol**	**Name**	**Function**	**Gene symbol**	**Name**	**Function**
CD160	CD160	Associated with peripheral blood NK cells and CD8 T lymphocytes with cytolytic effector activity	ADORA2A	Adenosine receptor A2a	Mainly expressed in the basal ganglia and immune tissues
CD86	T-lymphocyte activation antigen CD86	Receptor involved in the costimulatory signal essential for T-lymphocyte proliferation and interleukin-2 production	BTLA	B and T lymphocyte associated	A receptor that relays inhibitory signals to suppress the immune response
CTLA4	Cytotoxic T-lymphocyte-associated protein 4	Inhibitory receptor acting as a major negative regulator of T cell responses	IL2RA	Interleukin 2 receptor subunit alpha	Regulation of immune tolerance by controlling TREGs activity
IL10	Interleukin 10	Major immune regulatory cytokine that acts on many cells of the immune system	KLRC1	Killer cell lectin-like receptor C1	Recognition of MHC class I HLA-E molecules by NK cells and some cytotoxic T-cells
IL2RA	Interleukin 2 receptor subunit alpha	Regulation of immune tolerance by controlling regulatory T cells (TREGs) activity	LTA	Lymphotoxin alpha	Cytokine binding to TNFRSF1A/TNFR1, TNFRSF1B/TNFBR, and TNFRSF14/HVEM
IL6	Interleukin 6	Regulation of the final differentiation of B-cells into Ig-secreting cells	TMEM173	Transmembrane protein 173	Facilitator of innate immune signaling
LAG3	Lymphocyte activating 3	Inhibitory receptor on antigen-activated T-cells	TNFRSF17	TNF receptor superfamily member 17	Promotes B cell survival and plays a role in the regulation of humoral immunity
LTA	Lymphotoxin alpha	Cytokine binding to TNFRSF1A/TNFR1, TNFRSF1B/TNFBR, and TNFRSF14/HVEM	TNFRSF18	TNF receptor superfamily member 18	Regulation of CD3-driven T cell activation and programmed cell death
PDCD1LG2	Programmed cell death 1 ligand 2	Interaction with PDCD1 inhibits T cell proliferation	TNFSF13B	TNF superfamily member 13b	Stimulation of B and T cell function and the regulation of humoral immunity
TGFB1	Transforming growth factor beta 1	Regulates the growth and the differentiation of various cell types	TNFSF4	TNF superfamily member 4	Cytokine that co-stimulates T cell proliferation and cytokine production
TIGIT	T cell immunoreceptor with Ig and ITIM domains	Binds to PVR, decreases the secretion of IL12B, and suppresses T cell activation	TNFSF9	TNF superfamily member 9	Induces the proliferation of activated peripheral blood T-cells
TNFSF13B	TNF superfamily member 13b	Stimulation of B and T cell function and regulation of humoral immunity	VSIR	V-set immunoregulatory receptor	Immunoregulatory receptor inhibiting the T cell response
TNFSF14	TNF superfamily member 14	Delivers costimulatory signals to T cells, leading to T cell proliferation and IFNG production	KDR	Kinase insert domain receptor	Tyrosine-protein kinase that acts as a cell-surface receptor for VEGFA, VEGFC, and VEGFD
NECTIN2	Nectin cell adhesion molecule 2	Modulator of T cell signaling			

### Construction of Nomogram

**Figure 8:**
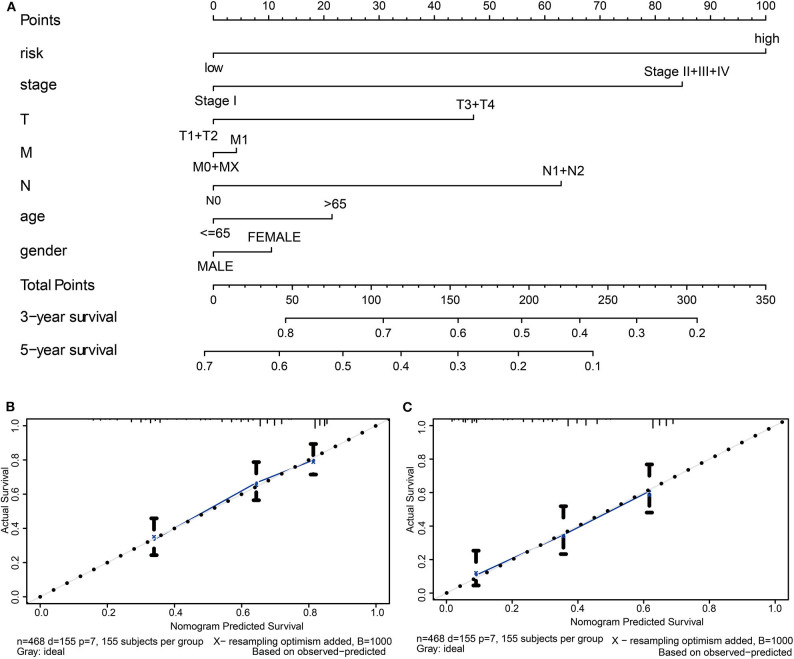
Establishment of the prognostic nomogram in LUAD with the inclusion of the risk score. **(A)** A nomogram for predicting 3- and 5-year survival possibilities of individual LUAD patients. The calibration curve of 3-year **(B)** and 5-year survival of LUAD patients **(C)**. The 45° dashed line represented a perfect uniformity between nomogram-predicted and real possibilities.

Finally, we constructed a prognostic nomogram in LUAD to anticipate the individuals' survival probability by weighing risk score, stage, T, N, M, age, and gender. Calibration was performed for the nomogram. The calibration curves showed that the nomogram-predicted probability (solid line) well-matched the idea reference line (dashed line) for the 3- and 5-year survival (Figure 8). We also appraised the nomogram's predictive discrimination by using a C-index that quantifies the level of agreement between the nomogram-derived probabilities and the actual observation of death. Our prognostic nomogram reached a C-index of 0.71.

## Discussion

Several antibodies targeting immune checkpoints to treat NSCLC have been approved by the FDA due to their remarkable efficacy. However, eradication of tumors by the tumor-specific CD8+ T cells is a complicated multi-step process, involving tumor antigen, antigen presentation, T cell activation, traffic to the tumor, overcoming local suppression, re-stimulation by tumor APC, and execution of tumor cells killing (30). The efficacy of immunotherapies might be compromised if any one of these critical steps failed. Therefore, significant challenges still exist in the discovery of more effective immunotherapeutic agents and the selection of patients suitable for the treatments. Biomarkers that can accurately signify the immune status and the prognosis of patients would be tremendously valuable in improving therapeutic decision making in NSCLC.

In this study, we found a close linkage between ACK1 and the immunity of NSCLC. The *ACK1* gene expression was associated with immune cell infiltration levels and immunomodulators. We successfully produced multiple-gene risk prediction signatures from an ACK1-associated immunomodulator using the stepwise Cox regression model. Ultimately, the prognostic values of the risk prediction signatures were validated by a prognostic nomogram.

We first evaluated the composition of intratumoral immune subsets of the individual patient since some immunotherapies were developed to regulate these cells. For instance, T lymphocyte subsets, such as CD8^+^, might be used as an indicator of response to immunotherapies (31, 32). CIBERSORT analysis revealed that the composition of 22 immune subsets in the tumor microenvironment was dramatically changed in both LUAD and LUSC when compared to normal tissues. This method has been increasingly applied to comprehensively explore the contribution of immune infiltrating cells in lung cancer from different respects of review (6–9). These findings suggested that the patterns of intratumoral infiltration of immune cells are associated with NSCLC prognosis. However, the acquirement of the landscape of the infiltrating immune cells of individual patients remains demanding in the clinical setting so far. It would be more feasible to discover molecular biomarkers that designate the immune status of patients.

Intriguingly, we found that ACK1, a putative oncogene, was associated with immune cell infiltration in lung cancer. To the best of our knowledge, this study provided the first evidence of linkage between ACK1 and tumor immunity. We found that *ACK1* gene copy numbers were inversely associated with the infiltration levels of B cell, CD8+ T cell, CD4+ T cell, macrophage, neutrophil, and dendritic cells in lung cancer. In detail, the ACK1 mRNA levels were inversely related to the abundance of most of the immune cell types, especially in LUSC. The association of ACK1 and immunity was verified by GSEA of 188 lung cancer cell lines and our RNA-seq data.

Using TCGA-LUAD data, a KEGG pathway analysis of ACK1-associated immunomodulators revealed that the PI3K-AKT pathway and the Ras signaling pathway might be involved in ACK1-mediated immune response. Previous studies reported that ACK1 could induce a PI3K-independent activation of AKT through phosphorylating AKT at tyrosine 176 (11, 33). The ACK1 blockade was found to suppress the MAPK signaling pathway previously (34). Following our findings, numerous articles have demonstrated that the AKT signaling pathway takes part in the control of tumor immune surveillance, inflammation, and immunomodulation (35–37). The implications of MAPK signaling in immunity and immunotherapy were also extensively investigated (37–40). A PD-L1 blocker (atezolizumab), in combination with a MEK inhibitor (cobimetinib), has shown favorable efficacy in colorectal patients (41). A number of clinical trials in melanoma are ongoing to investigate the clinical response to the sequential administration of MAPK signaling pathway inhibitors and antibodies that neutralize immune checkpoints (41). Taken together, it is biologically plausible to speculate that the ACK1 inhibitors may also be able to boost tumor immunity, thereby favoring the antitumor efficacy of immune checkpoint blockers.

The open access of public high-throughput gene expression data sets facilitates the discovery of potentially more reliable and robust lung cancer biomarkers. Many research teams have attempted to build gene expression-based signatures as predictors of prognosis in NSCLC (42–46). Prognosis immune signatures in cancer have been described previously (47, 48). Ascierto et al. discovered that the differential expression of immune genes in tumors was associated with breast cancer relapse by profiling the gene expression of breast cancer samples with microarray (48). The same study also constructed a prognostic signature with five immune genes, which was able to discriminate the patient subgroup at a significantly increased risk of relapse (48). Li et al. retrieved genome-wide gene expression profiles of 2,414 early-stage non-squamous NSCLC patient samples out of 19 public NSCLC cohorts. They developed a prognostic immune signature of the 25 gene pairs containing 40 distinct genes with the utilization of the immune-related genes acquired from the ImmPort database (https://immport.niaid.nih.gov/home). The prognostic immune signature was able to split patients with early-stage non-squamous NSCLC into high- and low-risk groups with significantly different overall survival. The immune signature reached a moderate prognostic accuracy (C-index = 0.64), which was further enhanced (C-index = 0.70) by the composition of clinical features and immune signature (47).

Consistently, with ACK1-associated immunomodulators, we established immune gene signatures for LUAD and LUSC separately. The risk scores derived from the gene signatures were significantly associated with survival in LUAD and LUSC. Most of the immune genes integrated into the prognostic signatures participate in the regulation of the activity of T cells, highlighting the significance of T cell-mediated immunity in lung cancer. Interestingly, the immune gene signatures for LUAD and LUSC shared only three genes (i.e., LTA, TNFSF13B, and IL2RA), suggesting the differences in immune microenvironment between the two histological types. Lastly, we constructed a nomogram for personalized prognosis prediction with a C-index of 0.71. Our results demonstrated that the risk scores derived from ACK1-associated immunomodulators were able to discriminate risk groups defined by a differential expression of a set of signature genes. Our findings may accelerate the development of well-verified signatures for cancer prognoses.

Despite some merits of the current study, limitations should be addressed. First, all the analyses were conducted with public datasets. The findings need to be validated in the in-house patient population. Second, the mechanisms underpinning ACK1-medicated tumor immunity and the prognostic values of immune signatures should be explored in the future.

In conclusion, our results suggested that ACK1 might also play a role in the control of tumor immune microenvironments. The prognostic signatures derived from ACK1-associated immumomodulators were independently predictive of overall survival in lung cancer. Prospective studies are called to verify the clinical application of the biomarker in the personalized management of NSCLC.

## Data Availability Statement

Publicly available datasets were analyzed in this study, these can be found in the Cancer Genome Atlas (https://portal.gdc.cancer.gov/).

## Ethics Statement

Ethical review and approval was not required for the study on human participants in accordance with the local legislation and institutional requirements. The patients/participants provided their written informed consent to participate in this study.

## Author Contributions

YL and HA processed the data and conducted the analyses. ML and MZ prepared all the tables and figures. JZ and JM conceived and wrote the manuscript. All authors approved the final manuscript.

## Conflict of Interest

The authors declare that the research was conducted in the absence of any commercial or financial relationships that could be construed as a potential conflict of interest.

## Supplementary Material

Supplementary Figure 1 The abundance of 22 infiltrating immune cell subsets in cancerous and healthy biopsies for TCGA-LUAD **(A)** and -LUSC **(B)** cohorts calculated by the CIBERSORT method.

Supplementary Figure 2 Kaplan-Meier curves described the association between survival and six tumor immune cells as well as the *ACK1*/*TNK2* gene via the TIMER web-based tool (cistrome.dfci.harvard.edu/TIMER/) for LUAD **(A)** and LUSC **(B)**.

Supplementary Figure 3 Boxplots of *ACK1*/*TNK2* expression levels in tumor and normal tissues, as indicated.
