# Supplementary figures and images for: Comprehensive Analysis of the Immune Implication of ACK1 Gene in Non-small Cell Lung Cancer
Source: Front Oncol. 2020 Jul 23;10:1132. doi: 10.3389/fonc.2020.01132 (PMC7390926; doi:10.3389/fonc.2020.01132)

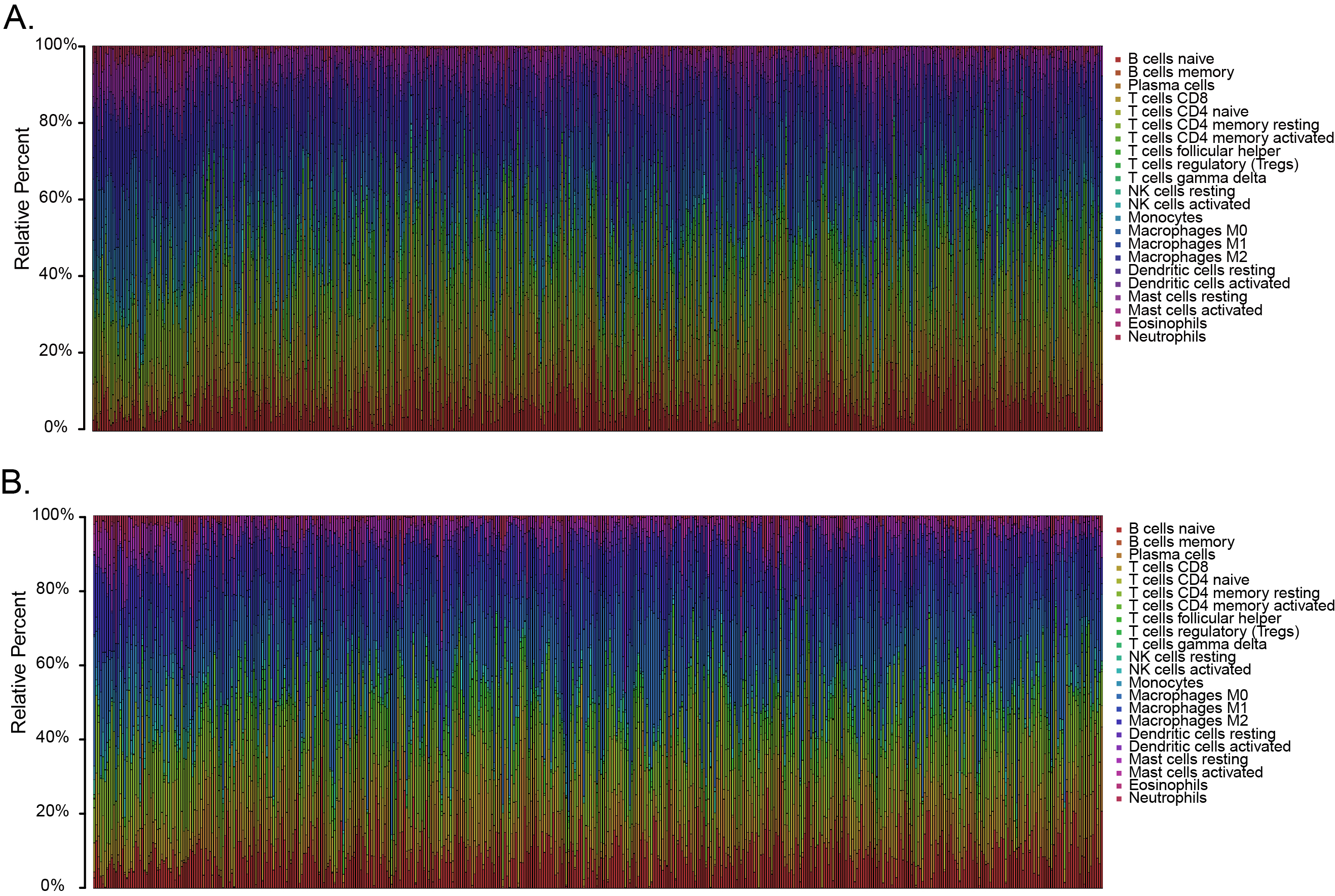

Supplement: Supplementary Figure 1 — The abundance of 22 infiltrating immune cell subsets in cancerous and healthy biopsies for TCGA-LUAD (A) and -LUSC (B) cohorts calculated by the CIBERSORT method. [file Image_1.JPEG]

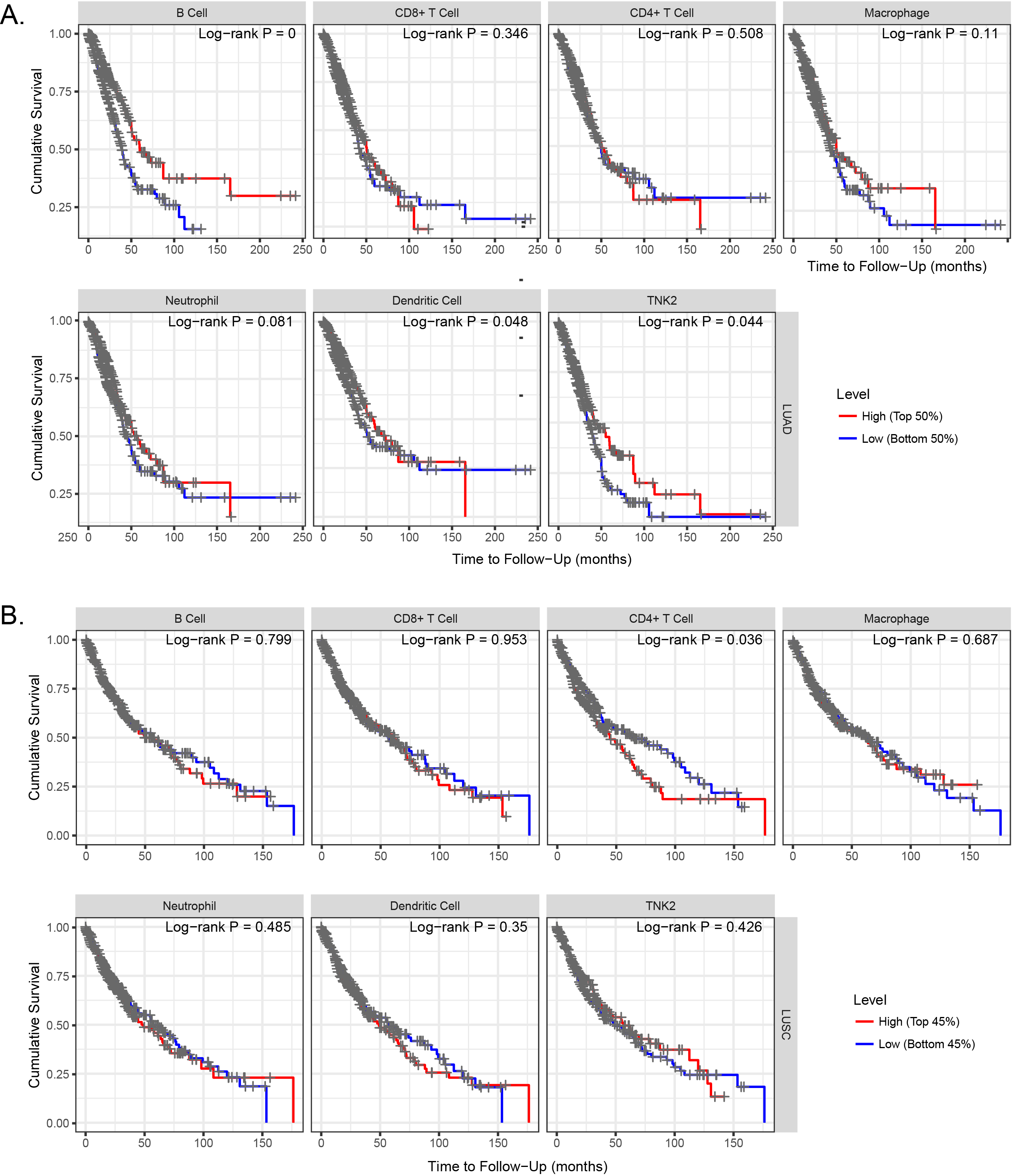

Supplement: Supplementary Figure 2 — Kaplan-Meier curves described the association between survival and six tumor immune cells as well as the ACK1/TNK2 gene via the TIMER web-based tool (cistrome.dfci.harvard.edu/TIMER/) for LUAD (A) and LUSC (B). [file Image_2.JPEG]

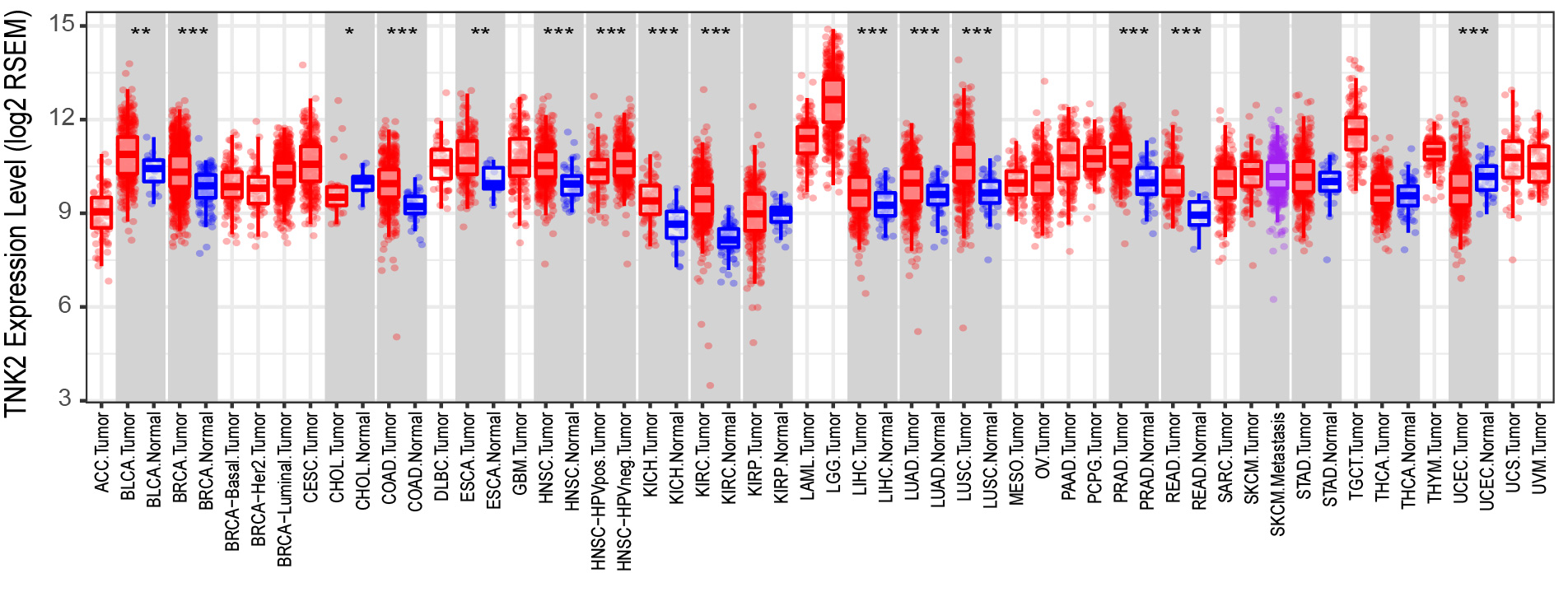

Supplement: Supplementary Figure 3 — Boxplots of ACK1/TNK2 expression levels in tumor and normal tissues, as indicated. [file Image_3.JPEG]
